# Additions to the *Xylariomycetidae* (*Sordariomycetes*) fungal flora from northern Thailand: four novel *Amphisphaeria* species

**DOI:** 10.3897/mycokeys.132.188390

**Published:** 2026-05-08

**Authors:** Milan C. Samarakoon, Ishara S. Manawasinghe

**Affiliations:** 1 Department of Entomology and Plant Pathology, Faculty of Agriculture, Chiang Mai University, Chiang Mai 50200, Thailand Beijing Key Laboratory of Environment Friendly Management on Fruit Diseases and Pests in North China, Institute of Plant Protection, Beijing Academy of Agriculture and Forestry Sciences Beijing China; 2 Center of Omics for High-Value Agriculture, Faculty of Agriculture, Chiang Mai University, Chiang Mai 50200, Thailand Center of Omics for High-Value Agriculture, Faculty of Agriculture, Chiang Mai University Chiang Mai Thailand; 3 Beijing Key Laboratory of Environment Friendly Management on Fruit Diseases and Pests in North China, Institute of Plant Protection, Beijing Academy of Agriculture and Forestry Sciences, Haidian District, Beijing 100097, China Department of Entomology and Plant Pathology, Faculty of Agriculture, Chiang Mai University Chiang Mai Thailand

**Keywords:** Agrobiodiversity, forest management, four new species, fungal diversity, taxonomy

## Abstract

In a continuing survey of xylarialean taxa with inconspicuous stromata, seven collections were obtained from twigs and branches at the late stage of senescence or the early stage of decomposition while attached to the host. These materials harbored xylarialean-like fungal structures and were collected during the wet seasons of 2022 and 2023 from forest and agroforestry fields in northern Thailand. Based on morphological examination and multigene phylogenetic analyses using maximum likelihood, maximum parsimony, and Bayesian inference of the internal transcribed spacer, partial 28S large subunit rDNA, partial RNA polymerase II second largest subunit, and partial β-tubulin genes, four new species, *Amphisphaeria longiostiolata*, *A. planibasalis*, *A. sanpakiaensis*, and *A. sribuabanensis*, are introduced. In addition, *A. hydei* on *Casearia* sp. and *A. oleae* on *Prunus* sp. are reported as new host records, and *A. pterocarpi* is documented as an additional collection from the region. All taxa are described and illustrated, with detailed morphological comparisons to phylogenetically related species, including taxa with and without available molecular data. In northern Thailand, *Amphisphaeria* species are predominantly recorded during the wet season, suggesting that seasonal conditions may influence sexual morph development. The recurrent occurrence of *Amphisphaeria* on woody substrates during this period suggests a potential endophytic phase and a shift in lifestyle within the genus. Further studies integrating endophytic surveys with ecological and nutritional data are needed to clarify the life history and ecological roles of *Amphisphaeria* species.

## Introduction

*Amphisphaeriaceae* was established by Winter (1887) and comprises predominantly saprobic species that are mainly involved in the decomposition of plant debris. However, a limited number of taxa exhibit hemibiotrophic or necrotrophic lifestyles (Shoemaker 1963; Wang et al. 2004; Senanayake et al. 2015). Hyde et al. (2024a) accepted only two genera within *Amphisphaeriaceae*, namely *Amphisphaeria* (= *Lepteutypa*) and *Labridella* (= *Griphosphaerioma*).

*Amphisphaeria* was introduced by Cesati and de Notaris (1863) to accommodate fungi characterized by superficial to semi-immersed, subglobose perithecial ascomata, occurring scattered or in small groups, with a conical ostiole. The ascomata possess a smooth, black, carbonaceous clypeus that is often inconspicuous; cylindrical, eight-spored asci with paraphyses; and ellipsoidal to oblong, one-septate ascospores that are blackish to deep brown or dark olive (Cesati and de Notaris 1863). By 2000, a total of 254 species had been accepted in *Amphisphaeria*, largely based on these morphological characters, although several species had already been transferred to other genera. Wang et al. (2004) re-examined 170 type specimens and recognized only 12 species as belonging to *Amphisphaeria*, transferring most of the remaining taxa to other genera due to the presence of bitunicate asci. Until 2015, only three additional species of *Amphisphaeria* had been introduced. Samarakoon et al. (2020) re-evaluated the morphology and phylogeny of *Amphisphaeria* and *Lepteutypa* and demonstrated that ascospore septation, previously used as a key character to distinguish *Lepteutypa*, is not a reliable criterion for generic delimitation, as both morphological observations and molecular phylogenetic analyses reveal no clear separation between the two genera. Consequently, *Lepteutypa* was synonymized under *Amphisphaeria* to resolve the taxonomic instability. However, ten *Lepteutypa* species lacking molecular data and asexual morphs were retained as ‘lepteutypa-like’ taxa, pending recollection to establish sexual–asexual connections, generate molecular data, and clarify their generic placement (Samarakoon et al. 2020).

In recent years, numerous new saprobic *Amphisphaeria* species have been described. Wang et al. (2023) reported the first endophytic member of *Amphis­phaeriaceae*, *A. orixae*, isolated from the roots of the medicinal plant *Orixa japonica* in China. Currently, 53 species are accepted in *Amphisphaeria*, of which 41 have been confirmed using molecular phylogenetic analyses (Chang et al. 2025; Tun et al. 2025; He et al. 2026).

Northern Thailand has contributed significantly to the collection and understanding of microfungal diversity in Thailand, revealing a high proportion of new species (Hyde et al. 2018). *Amphisphaeria* species have further enhanced our understanding of fungal diversity of *Xylariomycetidae*, with many new species described in recent years (Samarakoon et al. 2020; Tun et al. 2025; He et al. 2026). In an ongoing study on inconspicuous xylarialean taxa in northern Thailand, dead twigs and branches were collected during the rainy season. Based on morphological observations, detailed illustrations, and phylogenetic analyses, four new species and two new records of *Amphisphaeria* from northern Thailand are presented.

## Materials and methods

### Sample collection, isolation, and morphological studies

Fresh specimens were collected mainly during the wet seasons of 2022 and 2023 from forest and agroforestry fields in northern Thailand. For detailed characterization, external observations were carried out using a stereomicroscope (SteREO Discovery v8, Germany) attached to a camera (Axio Cam ERc5s), and microscopic photography was conducted using a Nikon DS-Ri2 camera connected to a Nikon ECLIPSE Ni (Tokyo, Japan) compound microscope. Where necessary, Melzer’s reagent was used to assess the chemical reaction of the apical ring, Congo red was used to improve visibility, and Indian ink was used to observe the mucilaginous sheath of ascospores. The photographs included in the figures were edited using Adobe Photoshop CS6 version 13.0 (Adobe Systems, USA) and measured using the Tarosoft® Image Framework program (v. 0.9.0.7).

The herbarium specimens were deposited in the Mae Fah Luang University Herbarium (MFLU), Chiang Rai, and the Sustainable Development of Biological Resources Research Herbarium, Department of Biology, Faculty of Science, Chiang Mai University (CMUB), Chiang Mai, Thailand. New taxa were linked with the MycoBank database (https://www.mycobank.org).

### DNA extraction, PCR amplification, and sequencing

Genomic DNA was extracted directly from fresh fruiting bodies using the PureDireX Genomic DNA Isolation Kit (BIO-HELIX Co., Ltd., Taiwan), following the manufacturer’s instructions. The internal transcribed spacer (ITS; primers ITS5/ITS4; White et al. 1990), partial 28S large subunit rRNA (LSU; LR0R/LR5; Vilgalys and Hester 1990), partial RNA polymerase II second largest subunit (*rpb*2; fRPB2-5F/fRPB2-7cR; Liu et al. 1999), partial β-tubulin (*tub*2; T1/T22; O’Donnell and Cigelnik 1997), and partial translation elongation factor 1-alpha (*tef*1-α; EF1-983F/EF1-2218R; Rehner and Samuels 1994) were amplified by polymerase chain reaction (PCR) using protocols described in Samarakoon et al. (2020). Each PCR reaction was performed in a total volume of 25 μL, comprising 12.5 μL of PCR Master Mix with dye (0.1 U Taq polymerase/μL, 500 μM of each dNTP, 20 mM Tris-HCl [*p*H 8.3], 100 mM KCl, and 3 mM MgCl_2_), 1 μL of each primer, 9.5 μL of nuclease-free double-distilled water, and 1 μL of genomic DNA template (25–50 ng). Amplified products were visualized by electrophoresis on 1% agarose gels using a D2000 DNA ladder (Realtimes Biotech, Beijing, China) and SafeView I nucleic acid staining dye (1 μL per 10 mL agarose). PCR products were stored at 4 °C prior to purification and sequencing. Purified PCR products were submitted to Apical Scientific SDN. BHD. (Seri Kembangan, Malaysia) for DNA sequencing.

### Phylogenetic analyses

Newly generated sequences were initially checked for quality and accuracy in BLASTn, and consensus sequences were assembled in SeqMan (DNAStar, Inc., Madison, WI, USA). Based on the BLASTn results and recent publications, related sequences for newly acquired sequences were downloaded from the NCBI (Table 1). The scoring matrix for nucleotide sequences and the 1.0 gap opening penalty settings of MAFFT v.7.036 (http://mafft.cbrc.jp/alignment/server/) were used to align individual loci using the FFT-NS-2 tree-based progressive method, with 20 PAM/*k* = 2. In BioEdit version 7.0 (Hall 1999), alignments were improved manually where necessary. Phylogenies were generated using maximum likelihood (ML), maximum parsimony (MP), and Bayesian inference (BI) approaches using individual (data not shown) and combined ITS–LSU–*rpb*2–*tub*2 alignments (Samarakoon et al. 2020). Newly generated *tef*1-α sequences were excluded from the phylogenetic analysis due to insufficient comparable data but were deposited in GenBank to support future research.

**Table 1.** Names, codes, and GenBank accession numbers of the taxa used in the phylogenetic analyses.

Taxa	Voucher/culture code	ITS	LSU	*rpb*2	*tub*2
*Amphisphaeria acericola*	MFLU 16-2479*	MK640423	MK640424	N/A	N/A
*A. acericola*	MFLUCC 14-0842*	MF614128	MF614131	N/A	N/A
*A. ailaoshanensis*	KUNCC 23-15520*	PP584673	PP584770	N/A	PQ046049
*A. ailaoshanensis*	KUNCC 23-15521*	PP584674	PP584771	N/A	PQ046050
*A. camelliae*	HKAS 107021*	MT756621	MT756615	MT789850	MT774368
*A. camelliae*	MFLU 20-0181*	MT756622	MT756616	MT789851	MT774369
*A. chiangmaiensis*	CMUB40017*	OR507139	OR507152	OR504416	N/A
*A. chiangmaiensis*	MFLU 23-0411*	OR507140	OR507153	N/A	N/A
*A. curvaticonidia*	MFLU 18-0789*	MT756623	MT756617	MT789852	N/A
*A. curvaticonidia*	HKAS 102288*	MT756624	MT756618	MT789853	N/A
*A. davidihuangtciae*	UESTCC: 25.0040*	PQ773395	PQ773415	PV075241	N/A
*A. davidihuangtciae*	UESTCC: 23.0548*	PQ773396	PQ773416	PV075242	N/A
*A. falcata*	CGMCC 3.23740*	OQ645270	OQ645284	OQ696281	OQ696283
*A. flava*	MFLU 18-0102*	MH971224	MH971234	N/A	MK033638
*A. fuckelii*	CBS 140409*	KT949902	KT949902	MK523280	MK523337
*A. fuckelii*	WU 33555	KT949903	KT949903	N/A	N/A
*A. guizhouensis*	GMBC 5901*	PV891981	PV891983	PV879933	PV879935
*A. guizhouensis*	GMBC 5902*	PV891982	PV891984	PV879934	PV879936
*A. guttulata*	MFLUCC 22-0052*	OQ101582	OQ101583	N/A	N/A
*A. hibiscicola*	HKAS 136910*	PQ570847	PQ570865	N/A	N/A
*A. hongheensis*	GMB 1135*	PQ165969	PQ166525	PQ249402	PQ249400
*A. hongheensis*	MHZU 24-0515*	PQ165968	PQ166524	PQ249401	PQ249399
*A. hydei*	CMUB40016*	OR507141	OR507154	OR504417	OR519975
*A. hydei*	MFLU 23-0412*	OR507142	OR507155	OR504418	OR519976
***A. hydei***	**MFLU 26-0123**	**PX568893**	**PX568900**	**PX513821**	**PX513834**
*A. hydeimucosa*	HKAS 132411*	PQ189779	PQ184734	PQ379998	PQ432241
*A. hydeimucosa*	UESTCC: 23.0462*	PQ191046	PQ184721	PQ379951	PQ432242
*A. karsti*	GZAAS 20-0147*	OR224991	OR209622	N/A	N/A
*A. karsti*	GZAAS 20-0148*	OR224992	OR209623	N/A	N/A
*A. kunmingensis*	KUNCC 23-15522*	PP584675	PP584772	N/A	PQ046051
*A. kunmingensis*	KUNCC 23-15523*	PP584676	PP584773	N/A	PQ046052
***A. longiostiolata***	MFLU 26-0120*	**PX568890**	**PX568897**	**PX513818**	**PX513831**
*A. magna*	HKAS 130270*	PP584677	PP584774	N/A	N/A
*A. magna*	HKAS 130271*	PP584678	PP584775	N/A	N/A
*A. mangrovi*	NFCCI-4247*	MG844283	MG844275	N/A	N/A
*A. mesuae*	MFLUCC 25-0197*	PV393830	PV299568	N/A	N/A
*A. micheliae*	HKAS 107012*	MT756625	MT756619	MT789854	MT774370
*A. micheliae*	MFLUCC 20-0121*	MT756626	MT756620	MT789855	MT774371
*A. mimusopis*	MFLUC 25-0076*	PV366837	PV299569	N/A	N/A
*A. neoaquatica*	MFLUCC 14-0045*	MK828607	MK835805	N/A	N/A
*A. oleae*	UESTCC: 23.0120*	OR253157	OR253314	OR253757	OR266103
*A. oleae*	CGMCC 3.24959*	OR253156	OR253313	OR253756	OR266102
***A. oleae***	**MFLU 26-0124**	**PX568894**	**PX568901**	**PX513822**	**PX513835**
*A. orixae*	GZCC 22-2031*	OQ064541	OQ064543	N/A	N/A
*A. orixae*	GZCC 22-2032*	OQ064542	OQ064544	N/A	N/A
*A. paraserianthis*	MFLU 25-0075*	PV393832	PV393833	N/A	N/A
*A. parvispora*	MFLU 18-0767*	MW240644	MW240574	MW658631	MW775601
***A. planibasalis***	MFLU 26-0119*	**PX568889**	**PX568896**	**PX513817**	N/A
*A. pseudomicheliae*	MFLU 25-0074*	PV393834	PV299570	N/A	N/A
*A. pterocarpi*	MFLU 25-0073*	PV366837	PV299564	N/A	N/A
*A. pterocarpi*	MFLUCC 25-0195*	PV366836	PV299566	N/A	N/A
***A. pterocarpi***	**MFLU 26-0125**	**PX568895**	**PX568902**	**PX513823**	**PX513836**
*A. qujingensis*	KUMCC 19-0186*	MN707568	MN707566	N/A	N/A
*A. qujingensis*	KUMCC 19-0187*	MN477033	MN556316	N/A	N/A
*A. sambuci*	CBS 131707*	KT949904	KT949904	MH554911	MH704632
*A. sambuci*	WU 33557	KT949905	KT949905	N/A	N/A
***A. sanpakiaensis***	MFLU 26-0121*	**PX568891**	**PX568898**	**PX513819**	**PX513832**
*A. schimae*	MFLUCC 25-0196*	PX488288	PV299567	N/A	N/A
*A. schimae*	MFLU 25-0071	PX488290	PV299565	N/A	N/A
*A. shangrilaensis*	HKAS 130272*	PP584679	PP584776	N/A	N/A
*A. shangrilaensis*	HKAS 130273*	PP584680	PP584777	N/A	N/A
***A. sribuabanensis***	MFLU 26-0122*	**PX568892**	**PX568899**	**PX513820**	**PX513833**
*A. sorbi*	MFLUCC 13-0721*	KR092797	KP744475	N/A	N/A
*Amphisphaeria* sp.	UESTCC: 01.0289	PV383357	PV368370	N/A	N/A
*Amphisphaeria* sp.	KoLRI_053241	MZ855365	N/A	N/A	N/A
*A. thailandica*	MFLU 18-0794*	MH971225	MH971235	MK033640	MK033639
*A. umbrina*	HKUCC 994	AF009805	AF452029	N/A	N/A
*A. umbrina*	PRA-JV24328	OL396664	N/A	N/A	N/A
*A. uniseptata*	CBS 114967*	MH553979	MH554197	MH554878	MH554638
*A. uniseptata*	HKAS 112607	OP377799	OP377898	OP473076	N/A
*A. verniciae*	UESTCC: 23.0122*	OR253155	OR253270	OR251140	OR266101
*A. verniciae*	CGMCC 3.24960*	OR253154	OR253269	OR251139	OR266100
*A. xishuangbannaense*	KUNCC 23-15524*	PP584681	PP584778	N/A	N/A
*A. xishuangbannaense*	KUNCC 23-15525*	PP584682	PP584779	N/A	N/A
*A. yunnanensis*	KUMCC 19-0188*	MN477177	MN556306	N/A	N/A
*A. yunnanensis*	KUMCC 19-0189	MN550997	MN550992	N/A	N/A
*A. zhaotongensis*	GMB 6412*	PX780726	PX780733	PX789093#	N/A
*A. zhaotongensis*	GMB 6413	PX780727	PX780732	PX789094#	N/A
*Clypeosphaeria mamillana*	CBS 140735*	KT949897	KT949897	MF489001	MH704637
*Cylindrium aeruginosum*	CBS 693.83	KM231854	KM231734	KM232430	KM232124
*C. algarvense*	CBS 124770*	MH863409	MH874925	N/A	N/A
*Neoarthrinium lithocarpicola*	CFCC 54456*	ON427580	ON427582	N/A	ON456914
*N. moseri*	CBS 164.80*	LN850995	LN851049	N/A	LN851154
*N. trachycarpi*	CFCC 53039*	MK301099	N/A	N/A	MK303395
*Xylaria hypoxylon*	CBS 122620*	AM993141	KM186301	KM186302	KM186300
*X. longipes*	CBS 148.73	MH860649	MH872351	KU684280	KU684204

Abbreviations: CBS: Westerdijk Fungal Biodiversity Institute, Utrecht, the Netherlands; CFCC: China Forestry Culture Collection Center, Research Institute of Forest Ecology, Environment and Protection, Beijing, China; CGMCC: China General Microbiological Culture Collection Center, Beijing, China; CMUB: Sustainable Development of Biological Resources Research Herbarium, Department of Biology, Faculty of Science, Chiang Mai University; GMB, GMBC: Guizhou Medical University, China; GZAAS, GZCC: Guizhou Academy of Agricultural Sciences, Guizhou, China; HKAS: Herbarium of Cryptogams, Kunming Institute of Botany, Academia Sinica, China; HKUCC: University of Hong Kong Culture Collection, Department of Ecology and Biodiversity, Hong Kong, China; KUMCC/KUNCC: Kunming Institute of Botany Culture Collection, China; MFLU, MFLUCC: Mae Fah Luang University, Chiang Rai, Thailand; MHZU: Mycological Herbarium of Zhongkai University of Agriculture and Engineering, China; NFCCI: National Fungal Culture Collection of India, Pune, India; PRA: The Herbarium of the Institute of Botany, Czech Academy of Sciences, Průhonice, Czech Republic; UESTCC: University of Electronic Science and Technology Culture Collection, Xiyuan, Chengdu, China; WU: The Herbarium of the University of Vienna, Austria. Type, authentic, and reference collections are denoted in ‘*’. ‘N/A’ indicates that the data are not available; ‘#’ signifies that it is not used for phylogeny.

The ML analysis was conducted using IQ-TREE (Nguyen et al. 2015; Trifinopoulos et al. 2016) with the rapid bootstrap algorithm using 1,000 bootstrap replicates. Substitution models were selected automatically. The MP analysis was performed in PAUP v.4.0b10 (Swofford 2002). Tree bisection reconnection (TBR) was employed as the branch-swapping algorithm, with maxtrees set to 1,000. Heuristic searches were carried out using 1,000 random sequence addition replicates. For MP analyses, tree statistics were calculated. Differences among resulting trees were assessed using the Kishino–Hasegawa test (Kishino and Hasegawa 1989). Bayesian inference (BI) analyses were performed using Markov chain Monte Carlo (MCMC) sampling in MrBayes v.3.1.2 (Huelsenbeck 2001; Zhaxybayeva and Gogarten 2002). Analyses were run for 1,000,000 generations with four chains and partitioned datasets, sampling every 100 generations. The first 2,500 trees, representing 25% of the total sampled trees, were discarded as burn-in. Posterior probabilities (PP) were calculated from the remaining 7,500 trees. Resulting phylogenetic trees were visualized using FigTree v.1.4.0 (Rambaut 2012), and final figures were prepared using Adobe Illustrator CS5 (version 15.0.0; Adobe, San Jose, CA, USA). All newly generated sequences were deposited in GenBank for future reference.

## Results

### Phylogenetic analyses

**Figure 1.**
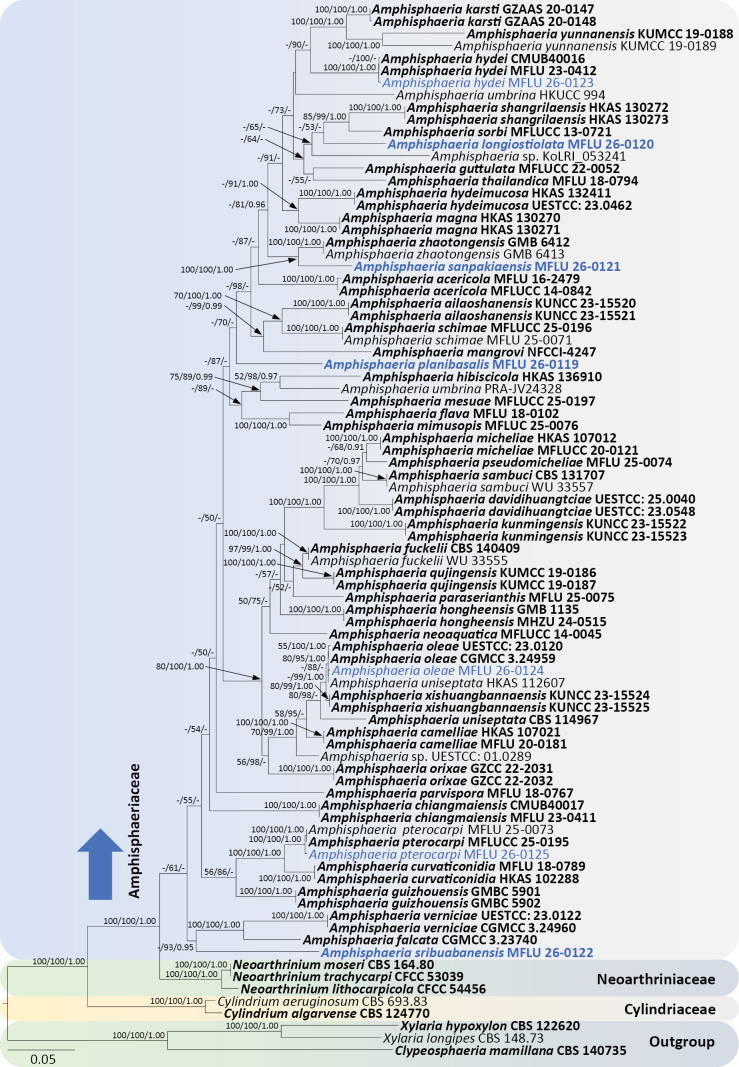
Phylogram generated from maximum likelihood analysis based on combined ITS–LSU–*rpb*2–*tub*2 sequence data. The tree is rooted in taxa from *Xylariaceae*. Bootstrap support values for MP and ML ≥50% and PP ≥0.9 are given above or below the nodes (MP/ML/PP). New collections are in blue. Type collections are in bold.

The combined ITS–LSU–*rpb*2–*tub*2 dataset comprised 86 sequences, including seven new sequences in this study. The phylogenetic tree was rooted using *Clypeosphaeria mamillana* (CBS 140735), *X. hypoxylon* (CBS 122620), and *X. longipes* (CBS 148.73). Two *rpb*2 sequences (PX789093 and PX789094) showed no significant similarity in BLASTn and exhibited high variability. Therefore, these sequences were excluded from the phylogenetic analyses. The concatenated alignment contained 3,443 characters, including gaps, with the following partitions: ITS (positions 1–719), LSU (720–1,576), *rpb*2 (1,577–2,643), and *tub*2 (2,644–3,443). Of these characters, 2,081 were constant or ambiguously constant (60.44%), 312 were parsimony-uninformative, and 1,050 were parsimony-informative, resulting in 1,666 distinct site patterns. Maximum parsimony (MP) analysis yielded a single most parsimonious tree with a tree length (TL) of 5,090, consistency index (CI) of 0.418, retention index (RI) of 0.663, relative consistency index (RC) of 0.277, and homoplasy index (HI) of 0.582. Maximum likelihood (ML) analysis with 1,000 bootstrap replicates recovered the best-scoring tree (Fig. 1) with a log-likelihood value of −26,940.3625. Estimated model parameters included a gamma shape parameter (α) of 0.5255 and substitution rates (*R*) of A–C = 1.0, A–G = 3.8372, A–T = 1.3512, C–G = 1.3512, C–T = 6.5367, and G–T = 1.0. Bayesian inference (BI) analysis showed satisfactory convergence, with the final average standard deviation of split frequencies falling below 0.01.

Phylogenetic analyses clearly separated *Amphisphaeriaceae* from *Neoarthriniaceae* (61% ML). Six major clades were resolved within the *Amphisphaeriaceae* lineage. *Amphisphaeria falcata*, *A. verniciae*, and MC22-101 formed an independent basal lineage with the remaining *Amphisphaeria* taxa, with weak support (55% ML). The seven new collections in this study were distributed among four *Amphisphaeria* clades. Collections MC22-106, MC22-111, and MC22-116 clustered with three previously described *Amphisphaeria* species with strong statistical support, whereas collections MC22-014, MC22-023, MC22-036, and MC22-101 formed distinct, well-separated lineages, indicating their independence from known taxa.

### Taxonomy

#### Amphisphaeria longiostiolata

Taxon classification

Fungi

AmphisphaerialesAmphisphaeriaceae

M.C. Samar. sp. nov.

320AFADE-6AE5-5560-9988-8E2A27856F27

862641

Fig. 2

##### Etymology.

The specific epithet “longiostiolata” refers to the long ostiolar neck.

**Figure 2.**
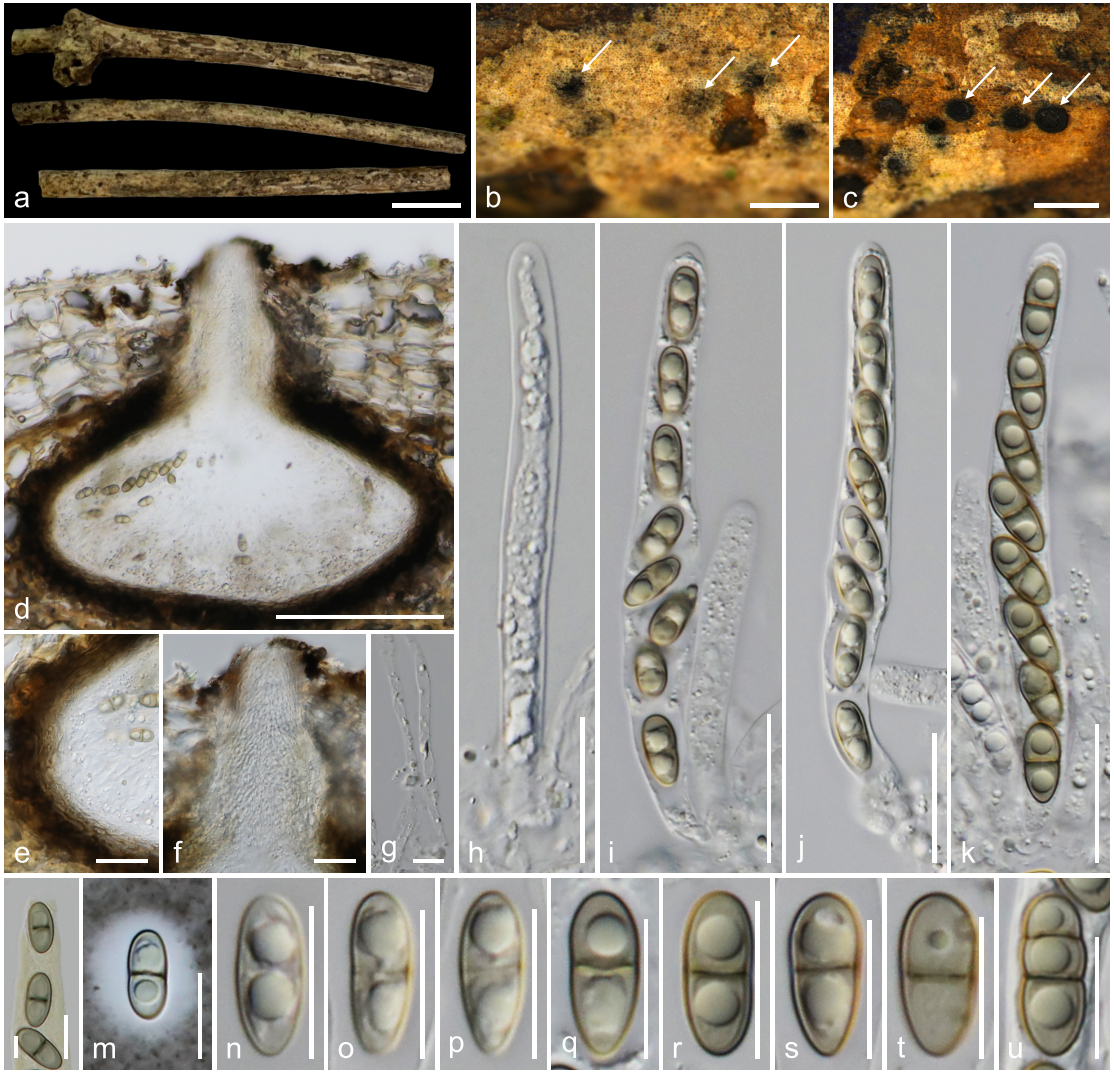
*Amphisphaeria longiostiolata* (MFLU 26-0120, holotype). **a**. Substrate; **b**. Ascomata in dead substrate (ascomata shown with white arrows); **c**. Horizontal section through ascomata; **d**. Vertical section through ascoma; **e**. Peridium; **f**. Vertical section through ostiolar canal; **g**. Paraphyses; **h–k**. Asci; **l**. Ascus in Melzer’s reagent; **m–u**. Ascospores (**m** in Indian ink). Scale bars: 1 cm (**a**); 500 μm (**b, c**); 100 μm (**d**); 20 μm (**e, f, h–k**); 10 μm (**g, l–u**).

##### Holotype.

MFLU 26-0120.

##### Description.

***Saprobic*** on an unidentified dicotyledonous dead branch. **Sexual morph**: ***Ascomata*** 200–260 × 200–240 μm (*x̄* = 238 × 223 μm, *n* = 5), immersed, visible as raised dark black-brown area, center black dots, solitary, scattered, in cross-section, globose to sub-globose. ***Ostioles*** centric, prominent, long (95–120 μm), conical ostiolar neck, ostiolar canal periphysate. ***Peridium*** 15.5–21 μm (*x̄* = 19.6 μm, *n* = 10) wide, multi-layered, outer layer thick, tightly attached to host tissue, comprising dark brown to black, thick-walled cells of ***textura angularis***, inner layer thin, composed of hyaline, thin-walled cells of ***textura angularis***. ***Paraphyses*** 3–5 μm (*x̄* = 4.1 μm, *n* = 20) wide, longer than asci, cellular, septate, branched, guttulate, embedded in a gelatinous matrix. ***Asci*** 80–100 × 6–9.5 μm (*x̄* = 89.5 × 7.6 μm, *n* = 20), eight-spored, unitunicate, cylindrical, J− in Melzer’s reagent, apically rounded. ***Ascospores*** 10.5–15.5 × 4–6.5 μm (*x̄* = 12.6 × 5 μm, *n* = 35), L/W 2.5, uniseriate, hyaline when young, light brown when mature, ellipsoid, one-septate (rarely two-septate), constricted at septa, bi-guttulate, smooth-walled, mucilaginous sheath 1.5–4.6 μm (*x̄* = 3.1 μm, *n* = 20). **Asexual morph**: Undetermined.

##### Material examined.

Thailand • Lamphun Province, Mueang District, on an unidentified dead dicotyledonous branch attached to the host, 22 October 2022, MC. Samarakoon, MC22-023 (**Holotype**MFLU 26-0120; **Isotype** CMUB40125).

##### Additional sequence.

PX513825 (*tef*1*-α*; MFLU 26-0120).

##### Notes.

In the combined ITS–LSU–*rpb*2–*tub*2 phylogeny, collection MC22-023 formed an independent clade sister to *A. shangrilaensis* and *A. sorbi* with robust statistical support (85% MP/99% ML/1.00 PP). The ITS sequence from MC22-023 is similar to that of *A. neoaquatica* (MFLU 15-0077; 92%, 6/452 gaps), *A. flava* (MFLU 18-0102; 92%, 11/461 gaps), *A. karsti* (GZAAS 20-0147; 91%, 6/459 gaps), and *A. shangrilaensis* (HKAS 130272; 87%, 25/616 gaps), while the LSU sequence is similar to that of *A. guttulata* (MFLU 22-0078; 96%, 6/934 gaps), *A. thailandica* (MFLU 18-0794; 96%, 7/927 gaps), *A. sambuci* (CBS 131707; 93%, 20/1170 gaps), and *A. fuckelii* (CBS 140409; 92%, 42/1195 gaps). The *rpb*2 and *tub*2 sequences of MC22-023 are similar to those of *A. hydeimucosa* (HKAS 132411; 87%, 0/1062 gaps and 87%, 26/1118 gaps), *A. hydei* (CMUB40016; 87%, 9/1014 gaps, and MFLU 23-0412; 86%, 30/1087 gaps), and *A. fuckelii* (CBS 140409; 85%, 5/1056 gaps and 90%, 10/825 gaps). The collection MC22-023 has solitary, immersed ascomata with a two-layered peridium and unitunicate asci, which are morphologically similar to those of other species in the genus. *Amphisphaeria sorbi*, *A. thailandica*, and *A. magna* possess apical ring J− in Melzer’s reagent, similar to MC22-023, and differ from *A. shangrilaensis* in having an apical ring J+ in Melzer’s reagent (Liu et al. 2015; Dissanayake et al. 2024). Compared to the similar taxa, collection MC22-023 has a distinct, long, conical ostiolar neck. *Amphisphaeria sorbi* and *A. magna* have larger ascomata compared to MC22-023 (350–380 × 450–505 μm and 300–350 μm × 450–520 μm vs. 200–260 × 200–240 μm). The species remain as ‘lepteutypa-like’, as ‘*Lepteutypa sabalicola*’ and ‘*L. tropicalis*’ possess immersed, subglobose ascomata and apical rings J− in Melzer’s reagent; however, short ostiolar neck, asci size, and appearance of ascomata differ from MC22-023 (Barr 1993; Dulymamode et al. 2001). Based on its distinct morphology and phylogeny, the new collection MC22-023 is introduced here as a new species, *A. longiostiolata*.

#### Amphisphaeria planibasalis

Taxon classification

Fungi

AmphisphaerialesAmphisphaeriaceae

M.C. Samar. sp. nov.

6065795F-71A8-5874-8CD2-63D523C530D8

862642

Fig. 3

##### Etymology.

The specific epithet “*planibasalis*” refers to the flat base of the ascomata.

**Figure 3.**
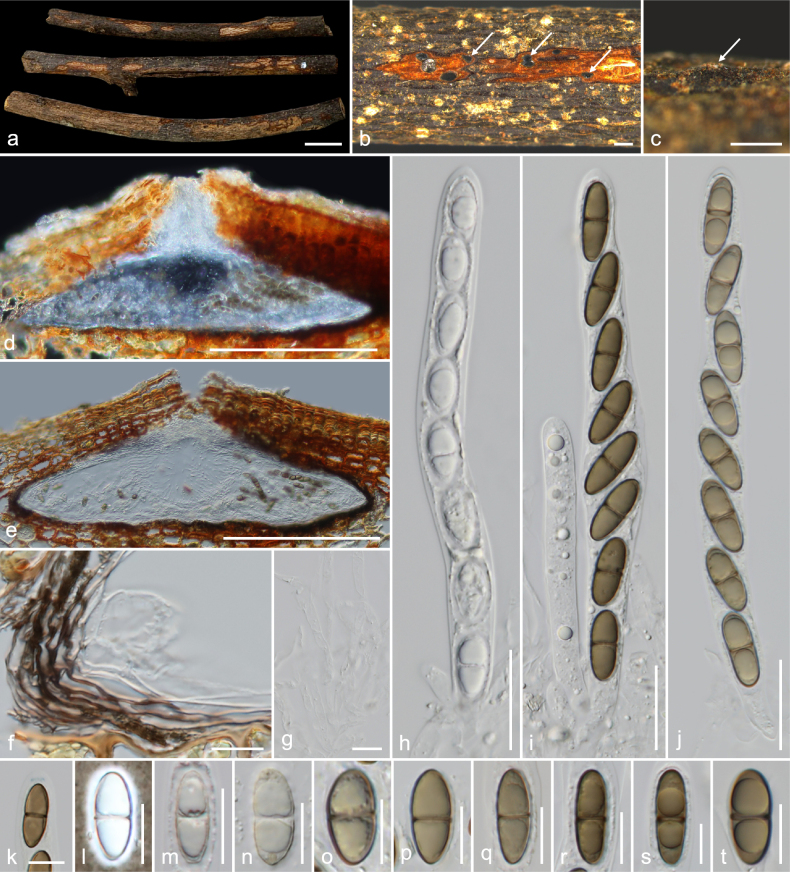
*Amphisphaeria planibasalis* (MFLU 26-0119, holotype). **a**. Substrate; **b, c**. Ascomata in dead substrate (ascomata shown with white arrows); **d, e**. Vertical sections through ascomata; **f**. Peridium; **g**. Paraphyses; **h–j**. Asci; **k**. Apical ring bluing in Melzer’s reagent; **l–t**. Ascospores (**l** in Indian ink). Scale bars: 1 cm (**a**); 500 μm (**b, c**); 200 μm (**d, e**); 20 μm (**h–j**); 10 μm (**f, g, k–t**).

##### Holotype.

MFLU 26-0119.

##### Description.

***Saprobic*** on an unidentified dicotyledonous dead branch. **Sexual morph**: ***Ascomata*** 345–450 × 120–235 μm (*x̄* = 416 × 183 μm, *n* = 7), immersed, visible as raised, black dots, solitary, rarely aggregated (two ascomata), scattered, in cross-section, conical with mostly flattened base. ***Ostioles*** centric, prominent, conical, ostiolar canal short and periphysate, 1.1–1.8 μm (*x̄* = 1.4 μm, *n* = 15). ***Peridium*** 6.5–10 μm (*x̄* = 8.5 μm, *n* = 10) wide, wider at the corners up to 18 μm, multi-layered, outer layer tightly attached to host tissue, comprising reddish brown, thick-walled cells of ***textura angularis***, inner layer composed of hyaline, thin-walled cells of ***textura angularis***. ***Paraphyses*** 2.7–5.3 μm (*x̄* = 3.6 μm, *n* = 20) wide, longer than asci, cellular, septate, branched, guttulate, embedded in a gelatinous matrix. ***Asci*** 110–145 × 8.5–14 μm (*x̄* = 127 × 11.2 μm, *n* = 20), eight-spored, unitunicate, cylindrical, with a 3–4 × 0.6–1.1 μm (*x̄* = 3.5 × 0.9 μm, *n* = 10), discoid, apical ring, J+ in Melzer’s reagent, apically rounded. ***Ascospores*** 13.5–19.5 × 5–7.5 μm (*x̄* = 16.2 × 5.8 μm, *n* = 35), L/W 2.8, uniseriate, hyaline when young, light to dark brown when mature, ellipsoid, one-septate, constricted at septum, prominent bi-guttulate when immature, smooth-walled, thin mucilaginous sheath 0.7–2 μm (*x̄* = 1.4 μm, *n* = 20). **Asexual morph**: Undetermined.

##### Material examined.

Thailand • Lamphun Province, Mueang District, on an unidentified dead dicotyledonous branch attached to the host, 22 October 2022, MC. Samarakoon, MC22-014 (**Holotype**MFLU 26-0119; **Isotype** CMUB40143).

##### Additional sequence.

PX513824 (*tef*1*-α*; MFLU 26-0119).

##### Notes.

In the combined ITS–LSU–*rpb*2–*tub*2 phylogeny, collection MC22-014 formed a distinct branch. The ITS sequence of MC22-014 is similar to that of *A. umbrina* (PRA-JV24328; 90%, 17/596 gaps), *A. flava* (MFLU 18-0102; 90%, 17/598 gaps), *A. ailaoshanensis* (KUNCC 23-15520; 90%, 24/575 gaps), and *A. fuckelii* (CBS 140409; 89%, 12/595 gaps), while the LSU is similar to that of *A. hydei* (CMUB40016; 98%, 1/896 gap), *A. guttulata* (MFLU 22-0078; 98%, 2/892 gaps), *A. mesuae* (MFLU 25-0069; 97.9%, 4/900 gaps), and *A. thailandica* (MFLU 18-0794; 97%, 1/906 gap). The *rpb*2 sequence shows similarity to *A. fuckelii* (CBS 140409; 86%, 0/1055 gap), *A. qujingensis* (KUMCC 19-0187; 86%, 0/1049 gap), and *A. thailandica* (MFLU 18-0794; 85%, 5/781 gaps). Collection MC22-014 is similar to other taxa by having immersed ascomata, ellipsoid ascospores, and a J+ apical ring in Melzer’s reagent (the apical ring of *A. hibiscicola* is not described) (Sun et al. 2025). *Amphisphaeria flava* and *A. umbrina* differ from MC22-014 by the presence of a yellow halo on the substrate around the neck area and a clypeus, respectively. *Amphisphaeria conica* (current name *A. umbrina*) was originally introduced as *Sphaeropsis conica* as the generic type, highlighting the conical shape of the ascomata and the flat base. The ascospores of *A. flava* possess a sheath similar to that of MC22-014. However, the prominent conical ascomata of MC22-014 differ from the globose to subglobose ascomata found in the other species. ‘*Lepteutypa hexagonalis*,’ which lacks molecular data, resembles MC22-014 in having immersed, conical-pyramidal ascomata with a flattened base that are triangular, occurring singly or in pairs, and possessing a J+, discoid apical ring (Goh and Hyde 1997). However, it differs in having larger ascomata (700–800 × 600–700 µm) and fusiform, three-septate ascospores with 6–7 longitudinal ridges (Goh and Hyde 1997). Based on morphology and phylogeny, collection MC22-014 is identified as a new species, *A. planibasalis*.

#### Amphisphaeria sanpakiaensis

Taxon classification

Fungi

AmphisphaerialesAmphisphaeriaceae

M.C. Samar. sp. nov.

EC700102-5E39-543C-8DB5-686C62415505

862643

Fig. 4

##### Etymology.

The specific epithet “*sanpakiaensis*” refers to the San Pa Kia Highland Agricultural Research Station in Chiang Mai Province, Thailand, where the holotype specimen was collected.

**Figure 4.**
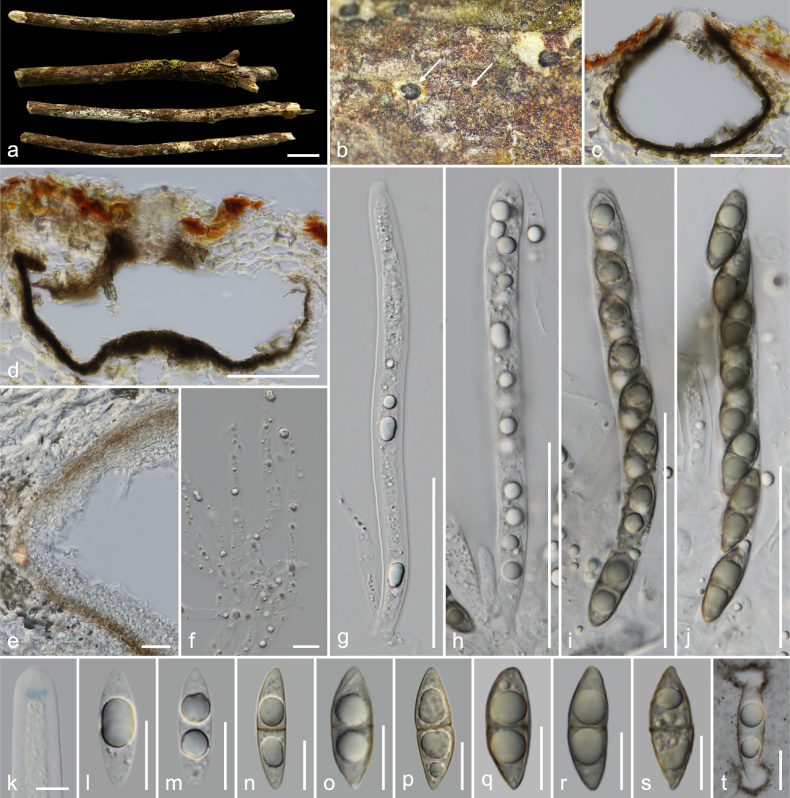
*Amphisphaeria sanpakiaensis* (MFLU 26-0121, holotype). **a**. Substrate; **b**. Ascomata in dead substrate (ascomata shown with white arrows); **c, d**. Vertical sections through ascomata; **e**. Peridium; **f**. Paraphyses; **g–j**. Asci; **k**. Apical ring bluing in Melzer’s reagent; **l–t**. Ascospores (**t** in Indian ink). Scale bars: 1 cm (**a**); 100 μm (**c, d**); 50 μm (**g–j**); 20 μm (**e**); 10 μm (**f, l–t**); 5 μm (**k**).

##### Holotype.

MFLU 26-0121.

##### Description.

***Saprobic*** on an unidentified dicotyledonous dead branch. **Sexual morph**: ***Ascomata*** 200–320 × 155–245 μm (*x̄* = 262 × 196.5 μm, *n* = 5), immersed, visible as black dots, solitary, scattered, in cross-section, globose to sub-globose. ***Ostioles*** centric, prominent, ostiolar canal filled with white amorphous tissues. ***Peridium*** 24–36 μm (*x̄* = 30.5 μm, *n* = 10) wide at immature stage, multi-layered, outer layer thin, tightly attached to host tissue, comprising brown, thick-walled cells of ***textura epidermoidea***, inner layer thick, composed of hyaline, thin-walled cells of ***textura epidermoidea***, 12–18 μm (*x̄* = 14.5 μm, *n* = 10) wide at mature stage, easy to disintegrate. ***Paraphyses*** 3.5–6.5 μm (*x̄* = 4.7 μm, *n* = 20) wide, longer than asci, cellular, septate, branched, guttulate, embedded in a gelatinous matrix. ***Asci*** 90–120 × 7.5–10 μm (*x̄* = 104 × 9.2 μm, *n* = 20), eight-spored, unitunicate, cylindrical, with a 3–3.7 × 1–1.5 μm (*x̄* = 3.4 × 1.3 μm, *n* = 7), discoid, apical ring, J+ in Melzer’s reagent, apically rounded. ***Ascospores*** 17.5–20.5 × 5.5–7.5 μm (*x̄* = 19 × 6.6 μm, *n* = 35), L/W 2.9, uniseriate, hyaline, aseptate with large central guttule when young, brown, ellipsoid with slightly pointed ends, one-septate, slightly constricted at septum, bi-guttulate, smooth-walled when mature, mucilaginous caps at both ends when immature. **Asexual morph**: Undetermined.

##### Material examined.

Thailand • Chiang Mai Province, Chiang Dao District, San Pa Kia Highland Agricultural Research Station, on an unidentified dicotyledonous dead branch attached to the host, 13 February 2023, MC. Samarakoon, MC22-036 (**Holotype**MFLU 26-0121; **Isotype** CMUB40145).

##### Additional sequence.

PX513826 (*tef*1*-α*; MFLU 26-0121).

##### Notes.

In the combined ITS–LSU–*rpb*2–*tub*2 phylogeny, MC22-036 forms an independent clade sister to *A. zhaotongensis* (100% MP/100% ML/1.00 PP). The ITS sequence is similar to that of *A. hydeimucosa* (HKAS 132411; 92%, 11/574 gaps), *A. ailaoshanensis* (KUNCC 23-15520; 90%, 15/567 gaps), *A. yunnanensis* (KUMCC 19-0188; 89%, 17/591 gaps), and *A. fuckelii* (CBS 140409; 88%, 27/599 gaps). The LSU sequence is similar to that of *A. guttulata* (MFLU 22-0078; 95%, 18/1160 gaps), *A. hydeimucosa* (HKAS 132411; 95%, 16/1147 gaps), and *A. schimae* (MFLU 25-0070; 95%, 17/1147 gaps). The *rpb*2 sequence is similar to that of *A. fuckelii* (CBS 140409; 87%, 0/1052 gaps), *A. qujingensis* (KUMCC 19-0187; 86%, 0/1049 gaps), and *A. hydeimucosa* (HKAS 132411; 85%, 3/1055 gaps), while the *tub*2 sequence is similar to *A. hydeimucosa* (HKAS 132411; 86%, 33/1089 gaps) and *A. hydei* (MFLU 23-0412; 86%, 35/1092 gaps). Collection MC22-036 possesses immersed ascomata, cellular, septate paraphyses, eight-spored, unitunicate cylindrical asci, and septate ascospores, which are morphologically similar to those of other species in the genus. However, MC22-036 has a ***textura epidermoidea*** peridium and ellipsoid, slightly pointed ends, one-septate, mature ascospores with mucilaginous caps at both ends, distinct from other *Amphisphaeria* species. The majority of the *Amphisphaeria* species have ***textura angularis*** cells in their peridium, and a few of them have ***textura intricata*** or ***textura prismatica***, but none of them have ***textura epidermoidea***. Based on its morphology and multigene phylogeny, the collection MC22-036 is introduced as *A. sanpakiaensis*.

#### Amphisphaeria sribuabanensis

Taxon classification

Fungi

AmphisphaerialesAmphisphaeriaceae

M.C. Samar. sp. nov.

924EC032-7B34-5BC0-B282-6555842C951D

862644

Fig. 5

##### Etymology.

The specific epithet “*sribuabanensis*” refers to the Sri Bua Ban Sub-District in Chiang Mai Province, Thailand, where the holotype specimen was collected.

**Figure 5.**
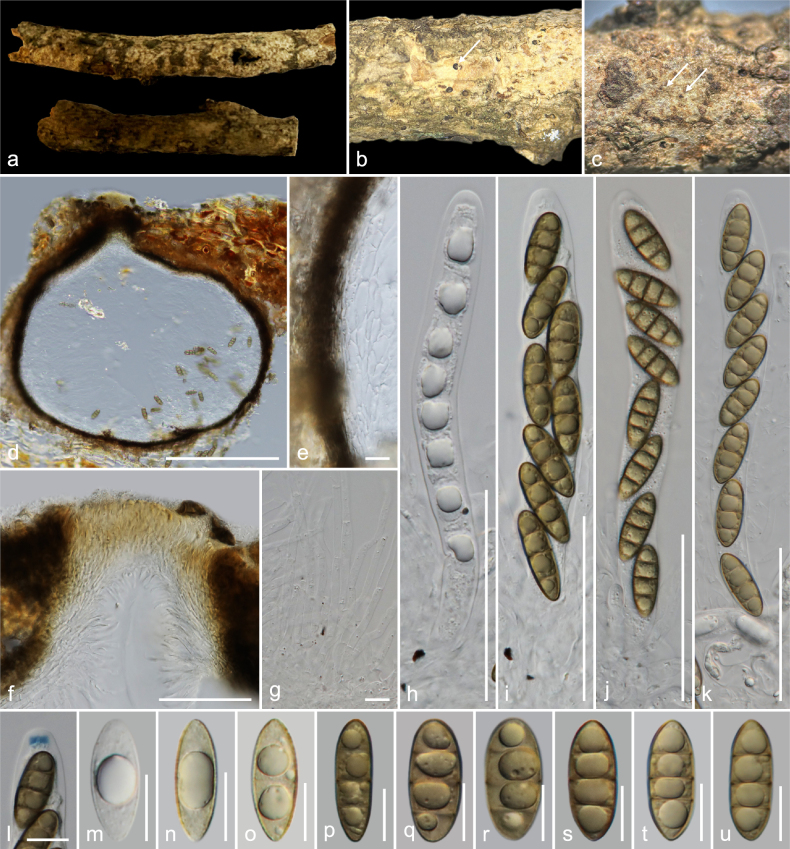
*Amphisphaeria sribuabanensis* (MFLU 26-0122, holotype). **a**. Substrate; **b, c**. Ascomata in dead substrate (ascomata shown with white arrows); **d**. Vertical section through ascoma; **e**. Peridium; **f**. Vertical section through ostiolar canal; **g**. Paraphyses; **h–k**. Asci; **l**. Apical ring bluing in Melzer’s reagent; **m–u**. Ascospores. Scale bars: 200 μm (**d**); 50 μm (**f, h–k**); 10 μm (**e, g, l–u**).

##### Holotype.

MFLU 26-0122.

##### Description.

***Saprobic*** on an unidentified dicotyledonous dead branch. **Sexual morph**: ***Ascomata*** 385–525 × 410–490 μm (*x̄* = 456 × 440 μm, *n* = 5), immersed, visible as dark brown to black dots, solitary, scattered, in cross-section, globose to sub-globose. ***Ostioles*** centric, prominent, ostiolar canal filled with periphyses, 1.3–3 μm (*x̄* = 1.6 μm, *n* = 15), bright yellow at the top. ***Peridium*** 15–28 μm (*x̄* = 21 μm, *n* = 10) wide, multi-layered, outer layer thin, tightly attached to host tissue, comprising dark brown, thick-walled cells of ***textura angularis***, inner layer thick, composed of light brown to hyaline, thin-walled cells of ***textura angularis***. ***Paraphyses*** 2.3–4.5 μm (*x̄* = 3.3 μm, *n* = 20) wide, longer than asci, cellular, septate, constricted at septa, branched, guttulate, embedded in a gelatinous matrix. ***Asci*** 140–175 × 12.5–19 μm (*x̄* = 158 × 14.7 μm, *n* = 20), eight-spored, unitunicate, cylindrical, with a 4.3–5 × 1.5–2 μm (*x̄* = 4.8 × 1.8 μm, *n* = 8), discoid, apical ring, J+ in Melzer’s reagent, apically rounded. ***Ascospores*** 19–26 × 6.5–11 μm (*x̄* = 22.2 × 8.6 μm, *n* = 35), L/W 2.6, uniseriate, hyaline, aseptate with large central guttule when immature, brown, ellipsoid, three-septate, constricted at median septum, guttulate, smooth-walled when mature. **Asexual morph**: Undetermined.

##### Material examined.

Thailand • Lamphun Province, Mueang District, Sri Bua Ban Sub-District, on an unidentified dicotyledonous dead branch attached to the host, 24 August 2023, MC. Samarakoon, MC22-101 (**Holotype**MFLU 26-0122; **Isotype** CMUB40146).

##### Additional sequence.

PX513827 (*tef*1*-α*; MFLU 26-0122).

##### Notes.

In the combined ITS–LSU–*rpb*2–*tub*2 phylogeny, MC22-101 forms a sister clade to the *A. verniciae* and *A. falcata* clade with strong statistical support (100% MP/100% ML/1.00 PP). The ITS, LSU, *rpb*2, and *tub*2 sequences of MC22-101 are similar to those of *A. fuckelii* (CBS 140409; 89%, 18/591 gaps; 93%, 17/1165 gaps; 12/1058 gaps; and 84%, 36/1016 gaps). The ITS sequence of MC22-101 is similar to that of *A. curvaticonidia* (MFLU 18-0789; 90%, 19/585 gaps) and *A. qujingensis* (KUMCC 19-0187; 89%, 17/591 gaps), while the LSU sequence is similar to that of *A. pterocarpi* (MFLU 25-0072; 94%, 15/1156 gaps), *A. guizhouensis* (GMBC 5901; 94%, 11/1144 gaps), and *A. schimae* (MFLU 25-0070; 94%, 13/1163 gaps). The *rpb*2 sequence of MC22-101 is similar to that of *A. parvispora* (MFLU 18-0767; 83%, 4/1051 gaps), *A. qujingensis* (KUMCC 19-0187; 82%, 12/1055 gaps), and *A. chiangmaiensis* (CMUB40017; 82%, 8/1012 gaps), while the *tub*2 sequence is similar to that of *A. camelliae* (HKAS 107021; 85%, 35/1014 gaps), *A. verniciae* (UESTCC: 23.0122; 89%, 5/762 gaps), and *A. micheliae* (HKAS 107012; 89%, 8/767 gaps). Collection MC22-101 has immersed ascomata, a multi-layered peridium, cellular, septate paraphyses, eight-spored, unitunicate, cylindrical asci, and septate ascospores, which are similar to those of the genus *Amphisphaeria*. Collection MC22-101 and *A. verniciae* share similar morphologies, having globose to subglobose, immersed ascomata; a peridium with ***textura angularis*** cells; brown, three-septate mature ascospores; and lacking mucilaginous sheaths (Li et al. 2024). However, MC22-101 has larger ascomata (456 × 440 μm vs. 150 × 170 µm), with bright yellow at the top of the ostiolar canal, larger asci (158 × 14.7 μm vs. 130 × 11 µm), and ascospores (22.2 × 8.6 μm vs. 17 × 6.5 µm). *Amphisphaeria verniciae* lacks information about the apical ring in Melzer’s reagent. *Amphisphaeria falcata* was described only from the asexual morph, which limits detailed morphological comparison (Chang et al. 2025). Several ‘*Lepteutypa*’ species have three-septate ascospores, which are similar to collection MC22-101. ‘*Lepteutypa sabalicola*’ and ‘*L. tropicalis*’ possess a J− apical ring in Melzer’s reagent and smaller ascomata compared to MC22-101 (Barr 1993; Dulymamode et al. 2001). ‘*Lepteutypa hederae*’ and MC22-101 share overlapping shapes and sizes of ascomata (500 × 400–800 μm vs. 456 × 440 μm, globose to subglobose), asci (130–190 × 15 μm vs. 140–175 × 12.5–19 μm), and ascospores (19–30 × 8–11 μm vs. 19–26 × 6.5–11 μm) and differ in having a J− apical ring in Melzer’s reagent (Rappaz 1995). ‘*Lepteutypa hexagonalis*’ has a conical-pyramidal, flattened base; triangular, larger ascomata (700–800 × 600–700 μm); ***textura intricata*** cells in the outer layer of the peridium; and fusiform ascospores with 6–7 longitudinal ridges (Goh and Hyde 1997). Based on the distinct morphology and phylogeny, collection MC22-101 is introduced as a new species, *A. sribuabanensis*.

#### Amphisphaeria hydei

Taxon classification

Fungi

AmphisphaerialesAmphisphaeriaceae

M.C. Samar., New Zealand J. Bot. 62(2–3): 262 (2024)

7E658ECD-6A60-5ADE-9B30-76E142849DD6

850826

Fig. 6

##### Description.

***Saprobic*** on a dead branch of *Casearia* sp. (*Salicaceae*). **Sexual morph**: ***Ascomata*** 230–280 × 175–215 μm (*x̄* = 264 × 198 μm, *n* = 5), immersed, visible dark brown to black dots, solitary, scattered, in cross-section, sub-globose to conical with mostly flattened base. ***Ostioles*** centric, prominent, ostiolar canal filled with periphyses, 1.3–2.2 μm (*x̄* = 1.7 μm, *n* = 15). ***Peridium*** 6–12 μm (*x̄* = 8.7 μm, *n* = 10) wide, multi-layered, outer layer thin, tightly attached to host tissue, comprising dark brown, thick-walled cells of ***textura angularis***, inner layer thick, composed of hyaline, thin-walled cells of ***textura angularis***. ***Paraphyses*** 2.5–4.5 μm (*x̄* = 3.4 μm, *n* = 20) wide, longer than asci, cellular, septate, constricted at septa, branched, guttulate, embedded in a gelatinous matrix. ***Asci*** 60–80 × 5.5–9 μm (*x̄* = 71.5 × 7 μm, *n* = 20), eight-spored, unitunicate, cylindrical, J− in Melzer’s reagent, apically rounded. ***Ascospores*** 10–16 × 3–5 μm (*x̄* = 12.2 × 3.9 μm, *n* = 35), L/W 3.1, uniseriate or 2-seriate, brown, ellipsoid, one-septate, constricted at the septum, guttulate, smooth-walled, lacking a mucilaginous sheath. **Asexual morph**: Undetermined.

**Figure 6.**
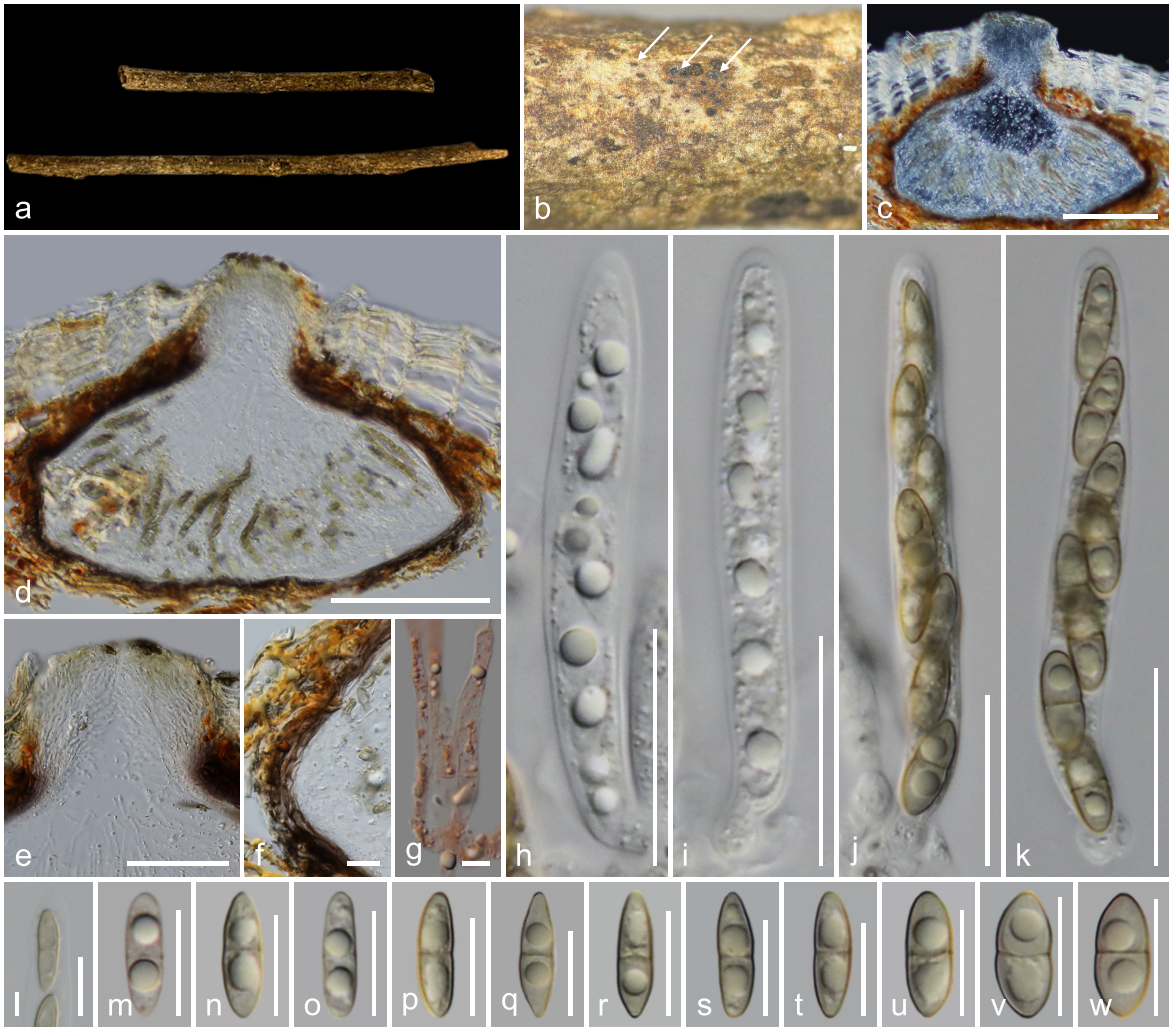
*Amphisphaeria hydei* (MFLU 26-0123). **a**. Substrate; **b**. Ascomata in dead substrate (ascomata shown with white arrows); **c, d**. Vertical sections through ascomata; **e**. Vertical section through the ostiolar canal; **f**. Peridium; **g**. Paraphyses (in Congo red); **h–k**. Asci; **l**. Ascus in Melzer’s reagent; **m–w**. Ascospores. Scale bars: 100 μm (**c, d**); 50 μm (**e**); 20 μm (**h–k**); 10 μm (**f, l–w**); 5 μm (**g**).

##### Material examined.

Thailand • Lamphun Province, Mueang District, Sri Bua Ban Sub-District, on a dead branch of *Casearia* sp. (*Salicaceae*) attached to the host, 24 August 2023, MC. Samarakoon, MC22-106 (MFLU 26-0123; CMUB40124).

##### Additional sequence.

PX513828 (*tef*1*-α*; MFLU 26-0123).

##### Notes.

In the combined ITS–LSU–*rpb*2–*tub*2 phylogeny, collection MC22-106 clustered with *A. hydei* with strong statistical support (100% ML). The ITS, LSU, *rpb*2, and *tub*2 sequences of MC22-106 showed 99% similarity to those of *A. hydei* CMUB40016 with 1/552, 0/933, 0/1005, and 0/1046 gaps, respectively. The ITS and LSU sequences of MC22-106 are similar to those of *A. yunnanensis*KUMCC 19-0188 (89%, 15/589 gaps and 97%, 3/944 gaps). The *rpb*2 and *tub*2 sequences of *A. hydeimucosa*HKAS 132411 show 86% (9/1052 gaps) and 87% (28/1063 gaps) similarity to MC22-106, respectively. *Amphisphaeria hydei* was originally described by Samarakoon (2024) from a dead branch. The new collection is morphologically similar to the type specimen in ascus shape and general dimensions but differs in having smaller asci (60–80 × 5.5–9 μm vs. 80–120 × 6–11 μm). Based on concordant morphological characters and high molecular sequence similarity, MC22-106 is identified as *A. hydei*. This collection represents the first record of *A. hydei* from *Casearia* sp. (*Salicaceae*) and is therefore reported here as a new host record.

#### Amphisphaeria oleae

Taxon classification

Fungi

AmphisphaerialesAmphisphaeriaceae

W. Li Li, R.R. Liang & Jian K. Liu, J. Fungi 10(3, no. 189): 10 (2024)

3C402932-B772-509B-B70F-9C876461EEDC

849633

Fig. 7

##### Description.

***Saprobic*** on a dead branch of *Prunus* sp. (*Rosaceae*). **Sexual morph**: ***Ascomata*** 540–640 × 250–295 μm (*x̄* = 591 × 275 μm, *n* = 5), immersed, visible as black dots, solitary, scattered, in cross-section, conical with mostly flattened base. ***Ostioles*** centric, prominent, wide, ostiolar canal filled with dense periphyses, 1–2 μm (*x̄* = 1.5 μm, *n* = 20). ***Peridium*** 12–20 μm (*x̄* = 16.4 μm, *n* = 10) wide, wider at the corners up to 27 μm, multi-layered, outer layer tightly attached to host tissue, comprising reddish brown, thick-walled cells of ***textura angularis***, easy to disintegrate when mature, inner layer thick, composed of hyaline, thin-walled cells of ***textura angularis***. ***Paraphyses*** 2.4–4.5 μm (*x̄* = 3.3 μm, *n* = 20) wide, longer than asci, cellular, septate, constricted at septa, branched, guttulate, embedded in a gelatinous matrix. ***Asci*** 105–155 × 6–8.5 μm (*x̄* = 130.5 × 7.2 μm, *n* = 20), eight-spored, unitunicate, cylindrical, with a 2.2–3 × 0.5–1.2 μm (*x̄* = 2.5 × 0.9 μm, *n* = 8), discoid, apical ring, J+ in Melzer’s reagent, apically rounded. ***Ascospores*** 14–19 × 5–6.5 μm (*x̄* = 16.2 × 5.8 μm, *n* = 35), L/W 2.8, uniseriate, brown, ellipsoid, one-septate, constricted at the septum, guttulate, smooth-walled. **Asexual morph**: Undetermined.

**Figure 7.**
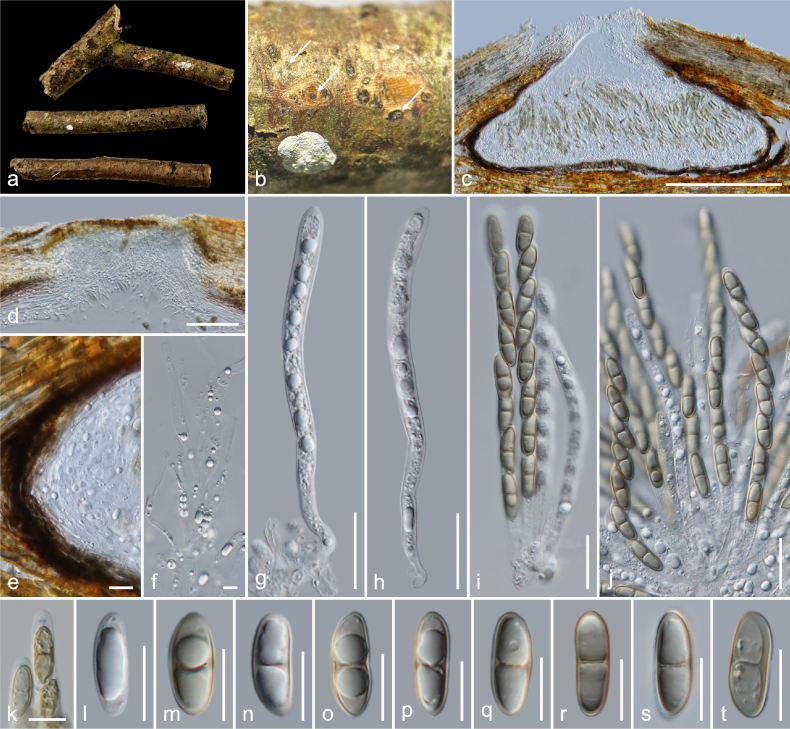
*Amphisphaeria oleae* (MFLU 26-0124). **a**. Substrate; **b**. Ascomata in dead substrate (ascomata shown with white arrows); **c**. Vertical section through ascoma; **d**. Vertical section through ostiolar canal; **e**. Peridium; **f**. Paraphyses; **g–j**. Asci; **k**. Apical ring bluing in Melzer’s reagent; **l–t**. Ascospores. Scale bars: 200 μm (**c**); 50 μm (**d**); 20 μm (**g–j**); 10 μm (**e, k–t**); 5 μm (**f**).

##### Material examined.

Thailand • Chiang Mai Province, Mae Rim District, Mae Raem, on a dead branch of *Prunus* sp. (*Rosaceae*) attached to the host, 17 October 2023, MC. Samarakoon, MC22-111 (MFLU 26-0124; CMUB40142).

##### Additional sequence.

PX513829 (*tef*1*-α*; MFLU 26-0124).

##### Notes.

In the combined ITS–LSU–*rpb*2–*tub*2 phylogeny, MC22-111 clustered with *Amphisphaeria oleae* and *A. xishuangbannaensis* with strong statistical support (100% ML). The ITS and LSU sequences from MC22-111 are similar to those of *A. oleae* (CGMCC 3.24959; 99%, 1/581 gap, 0/870 gaps), *A. uniseptata* (HKAS 112607; 100%, 0/566 gaps, 98%, 22/1185 gaps), and *A. xishuangbannaensis* (KUNCC 23-15524; 97%, 14/556 gaps, 99%, 6/694 gaps). The *rpb*2 sequence of MC22-111 is similar to that of *A. oleae* (CGMCC 3.24959; 99%, 0/1048 gaps), *A. uniseptata* (GZCC 20-0498; 100%, 0/987 gaps and CBS 114967; 99%, 0/832 gaps), and *A. camelliae* (HKAS 107021; 97%, 0/972 gaps). Collection MC22-111 is characterized by having immersed ascomata, eight-spored, unitunicate, cylindrical asci, and uniseriate, brown, ellipsoid ascospores, consistent with the genus *Amphisphaeria*. *Amphisphaeria oleae*, *A. xishuangbannaensis*, and MC22-111 have immersed ascomata visible as black dots but differ in shapes, having conical ascomata with mostly flattened bases versus globose to subglobose (Dissanayake et al. 2024; Li et al. 2024). Collection MC22-111 has a discoid apical ring and is J+ in Melzer’s reagent, similar to *A. oleae*, the type species introduced by Li et al. (2024) on branches of *Olea europaea* from China, but *A. xishuangbannaensis* has a J− apical ring in Melzer’s reagent. Following the similar morphologies and phylogenies, *A. oleae*, *A. uniseptata*, and *A. xishuangbannaensis* share close morphologies and phylogenies. However, based on the closest morphology and phylogeny, MC22-111 is taxonomically placed in *A. oleae*, and this is the first report of this species associated with *Prunus* sp. and the first report from Thailand.

#### Amphisphaeria pterocarpi

Taxon classification

Fungi

AmphisphaerialesAmphisphaeriaceae

Z.L. Tun & K.D. Hyde, MycoKeys 125: 1–31 (2025)

47CCC492-615B-5342-BCE5-1BDB1814A5EC

903750

Fig. 8

##### Description.

***Saprobic*** on an unidentified dicotyledonous dead branch. **Sexual morph**: ***Ascomata*** 320–430 × 155–205 μm (*x̄* = 373 × 176 μm, *n* = 5), immersed, visible black dots, solitary, scattered, in cross-section, oblate or depressed-globose. ***Ostioles*** centric, prominent, wide, ostiolar canal filled with dense periphyses, 1–2.2 μm (*x̄* = 1.6 μm, *n* = 20), occasionally ostiolar neck depressed when mature. ***Peridium*** 13–26 μm (*x̄* = 21 μm, *n* = 10) wide, multi-layered, comprising reddish brown, thick-walled cells of ***textura angularis***, inner layer thick, composed of hyaline, thin-walled cells of ***textura angularis***. ***Paraphyses*** 2.4–5.5 μm (*x̄* = 4.1 μm, *n* = 20) wide, longer than asci, cellular, septate, constricted at septa, branched, guttulate, embedded in a gelatinous matrix. ***Asci*** 95–150 × 10.5–15 μm (*x̄* = 126 × 12.5 μm, *n* = 20), eight-spored, unitunicate, cylindrical, with a 2.5–3.5 × 0.7–1.6 μm (*x̄* = 3.1 × 1.1 μm, *n* = 8), discoid, apical ring, J+ in Melzer’s reagent, apically rounded. ***Ascospores*** 18.5–22 × 5.5–7.5 μm (*x̄* = 20.5 × 6.7 μm, *n* = 35), L/W 3.1, uniseriate to overlapping biseriate, brown, ellipsoid, with 1 median, slightly constricted euseptum and two distosepta, guttulate, smooth-walled, mucilaginous sheath 1.3–2 μm (*x̄* = 1.6 μm, *n* = 15) when immature, thinner or lacking when mature. Asexual morph: Undetermined.

**Figure 8.**
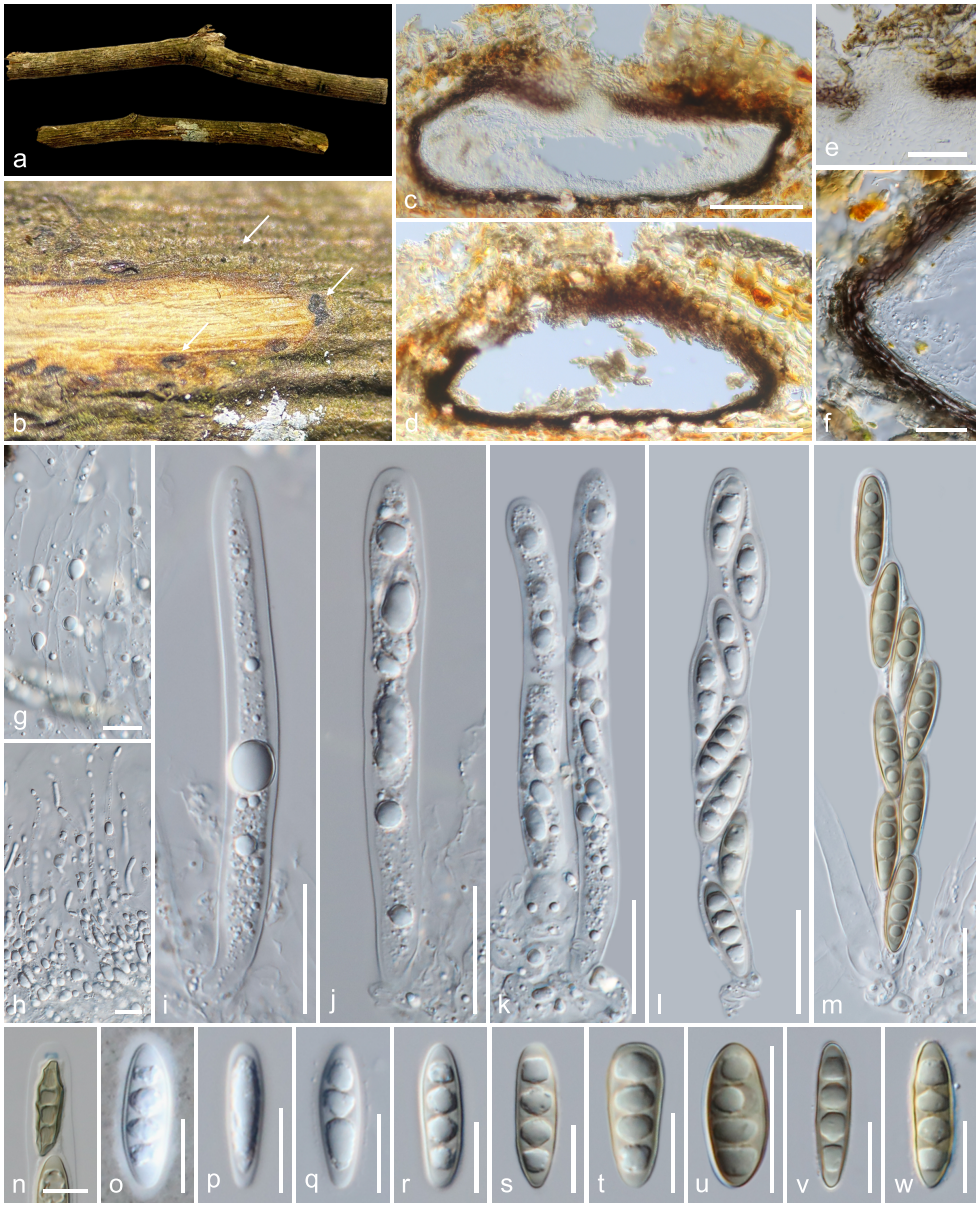
*Amphisphaeria pterocarpi* (MFLU 26-0125). **a**. Substrate; **b**. Ascomata in dead substrate (ascomata shown with white arrows); **c, d**. Vertical sections through ascomata; **e**. Vertical section through ostiolar canal; **f**. Peridium; **g, h**. Paraphyses; **i–m**. Asci; **n**. Apical ring bluing in Melzer’s reagent; **o–w**. Ascospores (**o** in Indian ink). Scale bars: 100 μm (**c, d**); 50 μm (**e**); 20 μm (**f, i–m**); 10 μm (**g, h, n–w**).

##### Material examined.

Thailand • Chiang Mai Province, Mae Rim District, Mae Raem, on an unidentified dicotyledonous dead branch attached to the host, 17 October 2023, MC. Samarakoon, MC22-116 (MFLU 26-0125; CMUB40144).

##### Additional sequence.

PX513830 (*tef*1*-α*; MFLU 26-0125).

##### Notes.

In the combined ITS–LSU–*rpb*2–*tub*2 phylogeny, MC22-116 clusters within the *A. pterocarpi* clade with strong support (98% ML). The ITS sequence of MC22-116 is similar to that of *A. pterocarpi* (MFLU 25-0072; 99%, 1/554 gap) and *A. curvaticonidia* (HKAS 102288; 93%, 28/578 gaps), while the LSU sequence shows close similarity to *A. pterocarpi* (MFLU 25-0072; 99%, 2/1062 gaps), *A. guizhouensis* (GMBC 5901; 97%, 7/1054 gaps), *A. schimae* (MFLU 25-0070; 94%, 15/1075 gaps), and *A. fuckelii* (CBS 140409; 94%, 17/1077 gaps). The *rpb*2 sequence is most similar to *A. curvaticonidia* (MFLU 18-0789; 95%, 0/882 gap) and *A. parvispora* (MFLU 18-0767; 86%, 2/1050 gaps). The *tub*2 sequence shows similarity to that of *A. fuckelii* (CBS 140409; 87%, 33/1036 gaps) and *A. hydeimucosa* (HKAS 132411; 89%, 13/839 gaps). However, the *tub*2 sequence also shows 89% similarity (13/839 gaps) to *Byssosphaeria vaginata*UESTCC: 23.0224 (acc. PP800890), a species placed in *Melanommataceae* (*Pleosporales*, *Dothideomycetes*) by Yu et al. (2025), indicating that this sequence may need re-evaluation. *Amphisphaeria pterocarpi* was introduced by Tun et al. (2025) from recently dead branches of *Pterocarpus rotundifolius* (*Fabaceae*) collected in Chiang Rai Province, Thailand. Collection MC22-116 shares the key morphological characteristics of the type specimen (MFLU 25-0073) of *A. pterocarpi* in having immersed, solitary ascomata; a peridium composed of ***textura angularis*** cells; eight-spored unitunicate cylindrical asci with a discoid apical ring that is J+ in Melzer’s reagent; and brown ellipsoid ascospores surrounded by a mucilaginous sheath. The new collection has oblate to depressed-globose ascomata, whereas the type specimen bears globose to subglobose ascomata, likely representing a difference in maturity or host substrate. Based on morphology and phylogeny, MC22-116 is identified as *A. pterocarpi*.

## Discussion

This study documents four new *Amphisphaeria* species, namely *A. longiostiolata*, *A. planibasalis*, *A. sanpakiaensis*, and *A. sribuabanensis*, together with two new host records: *A. hydei* on *Casearia* sp. and *A. oleae* on *Prunus* sp., and an additional collection of *A. pterocarpi* from northern Thailand. All taxa were collected from dead branches and twigs that were still attached to the host plant and at a late stage of senescence or an early stage of decomposition. This is an addition to the rapidly expanding species record in *Amphisphaeria*, as many of the other species have also come from historically understudied genera with only a few records, where targeted surveys plus DNA sequencing reveal hidden diversity (Phukhamsakda et al. 2022; Hyde et al. 2024b; Tun et al. 2025). The rapid increase in the number of *Amphisphaeria* species reported during the past decade is notable and prompts consideration of the factors underlying this pattern. This increase is most likely attributable to expanded sampling efforts and the application of molecular approaches for species identification, rather than a genuine ecological or evolutionary expansion (Wang et al. 2004; Raja et al. 2017; Samarakoon et al. 2020).

Seasonal conditions appear to influence both fungal abundance and the likelihood of detecting sexual morphs (Purhonen et al. 2017; Krah et al. 2023). Across ecosystems, fungal communities respond to changes in moisture, temperature, and host availability, leading to predictable seasonal shifts in diversity and composition. Studies from freshwater sediments, soils, and the atmosphere show that fungal assemblages vary with season, although local environmental factors often play a stronger role than season alone (Amma et al. 2018; Ji et al. 2021; Jurado et al. 2021; Chen et al. 2023). In northern Thailand, *Amphisphaeria* species are most frequently encountered during the wet season, suggesting that high humidity and sustained moisture may promote the initiation and development of fruiting structures (Samarakoon 2024; Tun et al. 2025). These observations indicate that the rainy season represents the most suitable period for collecting specimens with mature ascomata in this genus.

Fungal communities associated with senescing plant tissues differ markedly from those inhabiting living tissues. In woody plants, fungi isolated from living leaves are typically less diverse and compositionally distinct from those found in senesced leaves and woody litter, which support richer saprotrophic communities (U’Ren and Arnold 2016). Although some endophytes persist after senescence and may continue to function during decomposition, fungal assemblages reorganize as tissues age and break down. Culture-independent studies have further shown that fungal communities present before senescence are more similar to litter communities than to those in underlying soils, highlighting a gradual transition rather than a rapid shift (Liber et al. 2022). Members of *Xylariales* are commonly recovered as endophytes, and many ITS sequences in public databases remain unidentified or broadly assigned to xylaria-like taxa. In contrast, confirmed reports of endophytic *Amphisphaeria* remain rare (Wang et al. 2023). Most culture-based studies of endophytes report a strong dominance of *Ascomycota*, with *Sordariomycetes* frequently detected in aerial plant tissues of tropical and subtropical hosts (Tibpromma et al. 2022; Yadav and Meena 2025). Endophytic community structure varies with host species, tissue type, season, and sampling location, with leaves often showing higher colonization rates than woody tissues. Clear differences among tissues and seasons have been demonstrated in several host systems, including *Wrightia tinctoria* (Yadav and Meena 2025). Consideration of these variables may positively influence the isolation of additional *Amphisphaeria* species in their endophytic form.

The frequent recovery of *Amphisphaeria* sexual morphs from senescing twigs and branches, combined with the scarcity of confirmed endophytic records, raises questions about the nutritional ecology of this genus. It remains unclear whether *Amphisphaeria* species occur as endophytes during earlier stages of host tissue development or whether they primarily colonize hosts after senescence. Addressing this question will require targeted studies that examine both living and senescing tissues, ideally integrating culture-based approaches, high-throughput sequencing, and environmental data. Such work would help clarify whether *Amphisphaeria* species undergo lifestyle transitions and would provide a better understanding of their ecological roles within forest ecosystems.

## Supplementary Material

XML Treatment for Amphisphaeria longiostiolata

XML Treatment for Amphisphaeria planibasalis

XML Treatment for Amphisphaeria sanpakiaensis

XML Treatment for Amphisphaeria sribuabanensis

XML Treatment for Amphisphaeria hydei

XML Treatment for Amphisphaeria oleae

XML Treatment for Amphisphaeria pterocarpi

## Additional information

### Artificial Intelligence (AI) use

The authors accept full responsibility for the content of the manuscript, including the disclosure of any use of AI.

No AI tools were used in the preparation of this manuscript.

### Author ORCIDs

M.C. Samarakoon https://orcid.org/0000-0002-4815-125X

I.S. Manawasinghe https://orcid.org/0000-0001-5730-3596

### Data availability

All of the data that support the findings of this study are available in the main text.
